# PPARβ/δ priming enhances mesenchymal stromal cell anti-apoptotic properties on chondrocytes via ANGPTL4 and improves their therapeutic effects in osteoarthritis

**DOI:** 10.1093/stcltm/szag053

**Published:** 2026-08-05

**Authors:** Claudia Terraza-Aguirre, Rafael Contreras-López, Clara Bernard, Carolina Pradenas, Jholy De la Cruz, Audrey Barthelaix, Catalina Asencio, Gautier Tejedor, Cécile Boyer, Stéphanie Barrère-Lemaire, Christian Jorgensen, Mingxing Wei, Jerome Guicheux, Claire Vinatier, Farida Djouad

**Affiliations:** IRMB, Université de Montpellier, INSERM, Montpellier, 34295, France; CellVax, SAS, Villejuif, 94800, France; IRMB, Université de Montpellier, INSERM, Montpellier, 34295, France; IRMB, Université de Montpellier, INSERM, Montpellier, 34295, France; CellVax, SAS, Villejuif, 94800, France; IRMB, Université de Montpellier, INSERM, Montpellier, 34295, France; IRMB, Université de Montpellier, INSERM, Montpellier, 34295, France; IRMB, Université de Montpellier, INSERM, Montpellier, 34295, France; IRMB, Université de Montpellier, INSERM, Montpellier, 34295, France; IRMB, Université de Montpellier, INSERM, Montpellier, 34295, France; Nantes Université, Oniris, CHU Nantes, Inserm, Regenerative Medicine and Skeleton RMeS, UMR1229, Nantes F-44000, France; IGF, Université de Montpellier, CNRS, INSERM, Montpellier, 34094, France; IRMB, Université de Montpellier, INSERM, Montpellier, 34295, France; Clinical Immunology and Osteoarticular Disease Therapeutic Unit, Department of Rheumatology, CHU Montpellier, Montpellier, 34095, France; CellVax, SAS, Villejuif, 94800, France; Nantes Université, Oniris, CHU Nantes, Inserm, Regenerative Medicine and Skeleton RMeS, UMR1229, Nantes F-44000, France; Nantes Université, Oniris, CHU Nantes, Inserm, Regenerative Medicine and Skeleton RMeS, UMR1229, Nantes F-44000, France; IRMB, Université de Montpellier, INSERM, Montpellier, 34295, France; Clinical Immunology and Osteoarticular Disease Therapeutic Unit, Department of Rheumatology, CHU Montpellier, Montpellier, 34095, France

**Keywords:** mesenchymal stromal cells, PPAR-β/δ, osteoarthritis, anti-apoptotic effect, ANGPTL4

## Abstract

**Background:**

Osteoarthritis (OA) is a degenerative joint disease characterized by cartilage degradation, chondrocyte apoptosis, and inflammation. Mesenchymal stromal cells (MSCs) offer therapeutic potential, but their efficacy is hindered by oxidative stress and functional heterogeneity. We investigated whether PPARβ/δ priming enhances MSC therapeutic properties and explored the underlying mechanisms.

**Methods:**

MSCs were primed with the PPARβ/δ agonist GW501516. In a collagenase-induced OA mouse model, we assessed cartilage integrity by confocal laser scanning microscopy and histological grading. In vitro, we evaluated the effects of primed MSCs on oxidative stress resistance, chondrocyte apoptosis, and immunomodulation using DNA fragmentation, co-culture assays, and RNA sequencing.

**Results:**

PPARβ/δ priming significantly enhanced MSC resistance to oxidative stress, preserving their survival under H_2_O_2_-induced conditions. In co-culture experiments, primed MSCs exhibited a superior chondroprotective effect, reducing apoptosis in stressed chondrocytes. In vivo, primed MSCs improved cartilage volume, thickness, and integrity, lowering OA severity scores. RNA sequencing identified ANGPTL4 as a key mediator of these effects, as its silencing abolished MSC-mediated chondroprotection.

**Conclusion:**

PPARβ/δ priming enhances the therapeutic potential of MSCs by increasing their resistance to oxidative stress and anti-apoptotic effects on chondrocytes, mediated by ANGPTL4. These findings suggest a promising strategy for optimizing MSC-based therapies for OA.

## Graphical Abstract

**Figure:**
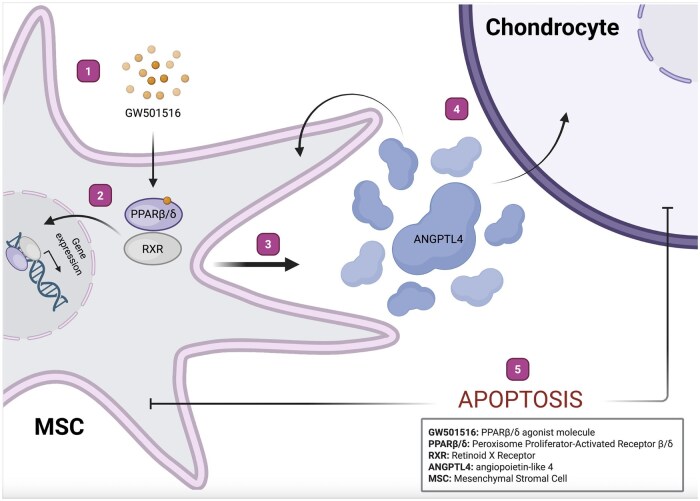
Created in BioRender. Djouad, F. (2026) https://BioRender.com/iaacnww.

Significance statement Mesenchymal stromal cells (MSCs) are a cornerstone of regenerative medicine, yet their clinical effectiveness in treating osteoarthritis (OA) is often hindered by the oxidative and inflammatory environment of the diseased joint. In this study, we present a novel priming strategy based on PPARβ/δ activation that significantly enhances the therapeutic performance of MSCs. By improving their resistance to oxidative stress and reinforcing their protective effects on cartilage cells, this approach not only boosts the regenerative capacity of MSCs but also addresses a critical limitation in current cell-based therapies. These findings open new perspectives for more effective and durable treatments of OA and may pave the way for broader applications of primed MSCs in other degenerative or inflammatory conditions.

## Introduction

Osteoarthritis (OA) is a widely prevalent degenerative joint disorder marked by cartilage breakdown, synovial inflammation, and subchondral bone remodeling, ultimately leading to chronic pain and disability.^1^ Current treatment options are mainly symptomatic, focusing on pain management and functional improvement but failing to address the underlying mechanisms or prevent disease progression.^2^ Consequently, there is a growing interest in developing innovative therapies that target the multifaceted pathophysiology of OA. In this context, Mesenchymal stromal cells (MSCs), especially those sourced from bone marrow, adipose tissue, and umbilical cord, have shown significant potential for OA treatment due to their immunomodulatory and tissue-protective properties.^3–5^

Thus, in clinical trials, following administration of MSCs, some studies suggested improvements in pain and joint function, while others reported little or no therapeutic benefit.^6^^,^^7^ These inconsistencies have been, in part, attributed to the poor *in vivo* survival rate and engraftment of MSC.^8^^,^^9^Another possible reason for these inconsistent results is their functional heterogeneity, which can be explained by the diversity of tissue sources (bone marrow, adipose tissue, and umbilical cord) used to isolate MSCs, the characteristics of the donor (such as age, gender, and state of health), and even the variability between isolated MSCs clones, amplification, and storage conditions.^9^^,^^10^ This heterogeneity inevitably leads to inconsistent therapeutic efficacy considerably limits their clinical use and could have repercussions on the essential functions of MSCs in the treatment of OA, such as their anti-apoptotic effect on chondrocytes, the only cell component of cartilage.

Chondrocyte apoptosis plays a significant role in the progression of OA. OA cartilage exhibits pronounced chondrocyte apoptosis, especially within the calcified layer, where numerous empty lacunae can be observed.^11^^,^^12^ This process of programmed cell death is characterized by morphological changes including DNA fragmentation, and chromatin condensation.^11^^,^^12^ Chondrocyte apoptosis in OA cartilage may be partially attributed to the degradation of the extracellular matrix (ECM), as the attachment of chondrocytes to collagen and proteoglycan (aggrecan) networks is essential for their survival.^13^

MSC derivatives such as extracellular vesicles (EVs) have shown promising results in reducing chondrocyte apoptosis. Specifically, human MSC-derived EVs can inhibit IL-1β-induced apoptosis in OA chondrocytes cultured *ex vivo.*^14^ However, it is essential to further investigate the anti-apoptotic mechanisms in the articular tissues of MSCs and their EVs, as this research could identify the mediators that promote the protection of articular cartilage in OA.

Peroxisome proliferator-activated receptors (PPARs) are nuclear receptors that play significant roles in various biological processes, including inflammation, lipid metabolism, cell growth, proliferation, and the maintenance of energy homeostasis within the body.^15–17^ Three isoforms of PPARs are recognized: PPARα (NR1C1), PPARβ/δ (NR1C2), and PPARγ (NR1C3).^18^ Due to their significant gene-regulatory effects, PPARs have long attracted the interest of researchers and clinicians alike. In particular, agonist targeting PPARβ/δ, significantly enhanced chondrogenesis and increased the expression of glycosaminoglycans and collagen type II in chondrocytes derived from MSCs.^19^ PPARβ/δ agonist combined with MSC-embedded scaffolds has shown promise as an effective therapeutic tool in a rabbit model of cartilage osteochondral defects.^20^ More recently, the influence of PPARβ/δ on the onset and development of OA was shown to improve ECM degradation and attenuated chondrocyte apoptosis via autophagy mediated by the AKT/mTOR signaling, providing protection against cartilage deterioration.^21^ Taken together, these studies indicate that activation of PPARβ/δ may serve to both stimulate hyaline cartilage formation and preserve its integrity.

Using murine MSCs, we have previously demonstrated that PPARβ/δ priming enhances their anti-apoptotic potential on cardiac cells *in vitro* and cardiac tissues *ex vivo.*^22^ In our study, we subjected cardiomyocytes and endothelial cells to H_2_O_2_-induced stress prior to co-culturing them with MSCs. We measured specific DNA fragmentation, a critical indicator of apoptosis, in these H_2_O_2_-stressed cells after their co-culture with either PPARβ/δ-primed or naïve MSCs. The use of PPARβ/δ priming to enhance the anti-apoptotic effects of MSCs has shown promise for both cardiomyocytes and endothelial cells.

Therefore, to optimize MSC therapeutic potential and bring them into routine clinical practice, the enhancement of MSC survival rate and engraftment in the damaged tissue as well as their anti-apoptotic properties on chondrocytes appears as a promising approach. In the pursuit of developing a standardized and effective MSC-based OA treatment, we investigated the role of PPARβ/δ in (1) enhancing MSC therapeutic potential in an OA experimental model and (2) promoting MSC survival and anti-apoptotic effects on chondrocytes. We also explored the mechanisms driving the enhanced properties of MSCs in response to PPARβ/δ priming.

In contrast to previous approaches that focused mainly on enhancing the immunomodulatory capacity of MSCs, here we propose a molecular priming strategy that aims to strengthen their survival and chondroprotective functions by activating the PPARβ/δ pathway and its downstream effector ANGPTL4.

## Methods

### Murine MSC isolation and amplification

Murine MSCs were isolated from bone marrow of 129/Sv mice (Charles River, France) and characterized as previously described.^23^ Briefly, bone marrow was flushed out of long bones. The cell suspension was plated and grown in a complete medium, consisting of DMEM high glucose supplemented with 10% fetal bovine serum (FBS), 2 mM L-Glutamine, and 1%P/S (Gibco, Thermofisher Scientific, USA). Non-adherent cells were removed. Murine MSC were grown at subconfluence and expanded at a density of 5.000 cells/cm^2^. Cells were used between passages 13 and 20.

### Human MSCs

Human MSCs derived from adipose tissue, bone marrow, and umbilical cord were harvested from patients who provided an informed, written consent. This study was conducted in accordance with ethical principles stated in the Declaration of Helsinki. Human MSC were cultured and used between passages 3 and 6.

### MSC stimulation

MSCs were plated in 6-well plates at a density of 15 000 cells/cm^2^ in complete medium. PPARβ/δ modulation was achieved by treating MSCs with 1 µM of a selective PPARβ/δ agonist (GW501516) (Sigma-Aldrich, USA) for 24 hours at 37 °C in a humidified 5% CO_2_ atmosphere.

### Collagenase-induced OA model

Animal experiments were in compliance with the guidelines and regulations of the Ethical Committee for animal experimentation of the Languedoc-Roussillon (approval 5349-2016050918198875). Final approval was granted by the French Ministry for Education, Higher Education and Research. This study adheres to the ARRIVE 2.0 guidelines. Mice were housed in a barrier-controlled environment (temperature: 20-25 °C; humidity: 40%-70%; light cycle: 12 hours/day), enriched with bedding, nesting and refuges materials, with unrestricted access to food and water. The collagenase-induced OA (CIOA) model was performed as previously described.^24^ Briefly, 10-week-old C57BL/6 mice (Charles River, France) were anesthetized with 2.5%-3% isoflurane, followed by intra-articular (IA) injection of 1U type VII collagenase (Sigma Aldrich, USA) in 5 μL saline into the knee joint on Days 0 and 2. On Day 7, groups of 15 mice received IA injections of either MSC_control_ or MSC_Ago_ (2.5 × 10^5^ cells/5 μL saline each group), or 5 μL saline for control groups. On Day 42, the mice were euthanatized in a CO_2_ chamber, and respiratory arrest and loss of consciousness were confirmed. Knee joints were then collected, fixed in 4% formaldehyde, and processed for further analysis.

### Confocal laser scanning microscopy

Articular cartilage of tibia medial plateau was scanned through their depth in XYZ-mode, with a confocal laser scanning microscope TCS SP8 MP (Leica, Germany) with a voxel size of 5 μm, a 5× dry objective and a UVlaser light source (405 nm). Stacks of images were then done and analyzed to quantitatively evaluate several parameters of articular cartilage. Assessment of cartilage morphometric parameters was performed in medial plateau of each tibia using Avizo^™^ software (FEI Visualization Sciences Group, France).

### Histological analysis

Samples were decalcified in a 5% formic acid solution for 2 weeks before being embedded in paraffin. Frontal sections of tibias were prepared by cutting 3 slices of 7 μm at 100 μm intervals, with the first section taken at 50 μm below the cartilage surface. The sections were stained with safranin O fast green. Cartilage degradation was quantified on lateral and medial plateau using the modified Pritzker OARSI score as previously described.^25^

### MSC siRNA transfection

MSC were transfected at 60% confluence with 50 nM of specific siRNA against *Angptl4*, (Dharmacon, UK) or negative control siRNA si-Scramble (Dharmacon, UK) using Oligofectamine reagent (Invitrogen, USA) following the manufacturer’s instructions. After overnight transfection, cells were washed with DPBS (Gibco) and cultured in complete medium.

### Immunosuppression assay

Spleens from 6 weeks old C57BL/6 mice were harvested and disaggregated to obtain a single-cell suspension. Red blood cells (RBCs) were lysed with RBC lysis buffer (Biolegend, USA), following manufacturer’s instruction. Splenocytes were labelled with Cell Trace Violet proliferation kit (Invitrogen, USA), and co-cultured with MSC_control_ or MSC_Ago_ at 1:10 ratio (MSC: Splenocytes) in mixed lymphocytes reaction (MLR) medium, composed of Iscove’s modified Dulbecco’s medium (IMDM) supplemented with 10% heat-inactivated fetal bovine serum 20 mM HEPES, 0.1 mM non-essential amino acids, 2 mM L-glutamine, 1 mM sodium pyruvate, 100 U/mL penicillin, 100 μm streptomycin (Gibco) and 50 μm β-mercaptoethanol (Life Technologies, Waltham, MA, USA). Splenocytes were activated with 2.5 µg/mL Concanavalin A (Sigma-Aldrich, USA). Cells were cultured at 37 °C in a humidified 5% CO_2_ atmosphere for 48 hours. Proliferation was assessed by FACS, and a viability marker (Life technologies) was used to remove dead cells from the analysis.

### Murine macrophage polarization

Bone marrow-derived monocytes were isolated from C57BL/6 mice by femoral flushing to obtain a single-cell suspension. Cells were cultured in 100 mm culture plates in MLR medium. Following a 4-hour incubation at 37 °C in a humidified 5% CO_2_ atmosphere, non-adherent cells were collected and seeded in a multi-well plate at the density of 2 × 10^5^ cells/cm^2^. MLR medium was supplemented with 20 ng/mL of macrophage colony-stimulating factor (M-CSF) (Miltenyi Biotec, Germany) on Days 0, 1 and 4. At Day 5, macrophages were pre-activated for 24 hours with 250 ng/mL lipopolysaccharides (LPS) (Sigma-Aldrich, USA) and 20 ng/mL interferon (IFN)-γ (R&D Systems, USA). At Day 6, differentiated macrophages were co-cultured in direct contact with MSC_control_ or MSC_Ago_ at 1:10 ratio (MSCs: Macrophages) in MLR medium. Cells were cultured for 24 hours at 37 °C in a humidified 5% CO_2_ atmosphere, until analysis.

### Flow cytometry

To assess macrophages polarization, cells were harvested using versene (Gibco) and incubated with 100 µM of Fc block (BD Bioscience) following the manufacturer’s indications. Then, cells were first stained using cell viability marker (Life technologies), and then labeled with specific antibodies against CD45 (clone 30-F11), CD11b (clone M1/70), CD11c (clone N418), CD38 (clone 90/CD38), CD80 (clone 16-10A1) (BD Bioscience), MHC-II (clone M5/114.15.2) (Miltenyi Biotec, USA), CD206 (clone C068C2) (Biolegend, USA), and F4/80 (clone BM8) (eBioscience, USA). Flow Cytometry analyses were performed on a BD FACSymphony A5 flow cytometer (BD Bioscience) equipped with BD FACSDiva software (version 9.0) (BD Bioscience). Data were analyzed using FlowJo software (v.10) (TreeStar Inc., USA).

### Synoviocyte co-culture

Human synoviocytes (SW982 cell line, ATCC, USA) were cultured in complete medium, under standard conditions. For co-culture assays, SW982 cells were plated in 24-well plates at 200.000 cells per well and pre-stimulated with 5 ng/mL of recombinant human IL-1β (R&D Systems, USA) for 6 hours in medium containing 2% FBS to induce a pro-inflammatory phenotype. IL-1β was maintained in the culture medium through the co-culture period. MSC_control_, MSC_Ago_, and MSC_siANGPTL4_ (prepared as described above) were seeded in 0.4 µm pore -size Transwell^®^ inserts (Falcon, USA) at density of 40.000 cells per insert and then placed over the synoviocytes monolayers (cell ratio of 1:5—MSC: SW982). Cells were co-cultured for additional 18 hours in the same medium. After co-culture, total RNA was extracted from SW982 cells as described below for downstream quantitative real-time polymerase chain reaction (qRT-PCR) analysis.

### Enzyme-linked immunosorbent assay

Supernatants from macrophage cultures were collected to quantify the production of tumor necrosis factor alpha (TNFα) and interleukin-10 (IL-10) using the DuoSet enzyme-linked immunosorbent assay (ELISA) Development kit (R&D Systems, USA) according to manufacturer’s instructions.

### DNA fragmentation assay

MSC_control_, MSC_Ago_, and MSC_siANGPTL4_ were cultured for 16 hours in assay medium containing 100 µM H_2_O_2_. The assay medium consisted of DMEM high glucose without FBS, supplemented with 2 mM L-Glutamine, and 1%P/S (Gibco, Thermofisher Scientific, USA).

Additionally, to assess the anti-apoptotic effect of MSC, a total of 100.000 TC28a2 chondrocytes cell line (ATCC, USA) was cultured in 24-well plates, alone or in the presence of MSC_control_, MSC_Ago_, or MSC_siANGPTL4_. TC28a2 and MSC were co-cultured separately using a transwell system 0.4 µm pore size (Falcon, USA), at a cell ratio of 1:2 (TC28a2: MSC) in assay medium containing 100 µM H_2_O_2_. As control for both cell types, 200 ng/mL of recombinant human ANGPTL4 (R&D Systems, USA) was added.

After H_2_O_2_ exposure of both MSCs and chondrocytes, DNA fragmentation was assessed using the cell death detection ELISA^PLUS^ kit (Roche, Switzerland), to quantify 180 bp soluble nucleosomes present in the cytoplasm as a measure of apoptosis. The assay was performed following the manufacturer’s instructions. Results were normalized to baseline conditions without H_2_O_2_.

### Quantitative real-time polymerase chain reaction

Total RNA was isolated from cells using the RNeasy mini kit (Qiagen, USA). RNA concentration and quality were evaluated with a NanoDrop OneC spectrophotometer. 500 ng of total RNA was reverse transcribed into cDNA using the SensiFAST^™^ Synthesis kit (Bioline, Meridian Life Science Company, USA) according to the manufacturer’s instructions. qRT-PCR assays were performed using the SensiFAST^™^ SYBR^®^ No-ROX kit (Bioline, USA), with the following amplification conditions: initial denaturation 95 °C for 10 min (1 cycle), followed by 40 cycles of denaturation at 95 °C for 10 seconds, annealing at 64 °C for 10 seconds, and elongation at 72 °C for 20 seconds. The melting curve stage was performed at 95 °C for 15 seconds, 65 °C for 1 minute, and 95 °C for 15 seconds. Primer sequences (Eurofins, France) are listed in Table 1. Results were normalized to the expression of a housekeeping gene. The 2^-ΔΔCt^ method was selected for changes in the expression level compared to a specific condition.

**Table 1:** List of primer sequences used for qRT-analysis.

Gene	Sequence forward	Sequence reverse
**Murine**		
***Angptl4***	CTG GAC AGT GAT TCA GAG ACG C	GAT GCT GTG CAT CTT TTC CAG GC
***Rsp9***	GCT GTT GAC GCT AGA CGA GA	ATC TTC AGG CCC AGG ATG TA
**Human**		
***ACTB***	ACC TAT GGG ATC GTG GCT GT	CCC CTA CCC CAA CTT GAC TT
***ANGPTL4***	GCA GGA TCC AGC AAC TCT TC	GGT CTA GGT GCT TGT GGT CC
***CCL2***	TGA AGC TCG CAC TCT CGC CT	GGG GAA TGA AGG TGG CTG CT
***CCL5***	GCT TTG CCT ACA TTG CCC GC	GGG TTG GAG CAC TTG CCA CT
***IL1B***	TGG CTT ATT ACA GTG GCA ATG AGG AT	TCG GAG ATT CGT AGC TGG ATG CC
***IL8***	GAG AGT GAT TGA GAG TGG ACC AC	CAC AAC CCT CTG CAC CCA GTT T
***RSP9***	GAT TAC ATC CTG GGC CTG AA	ATG AAG GAC GGG ATG TTC AC
***TNFA***	AGC CCA TGT TGT AGC AAA CCC TC	TGG TTA TCT CTC AGC TCC ACG CCA

### 3′ Seq-RNA profiling

Murine bone marrow MSC and human MSC from bone marrow, adipose tissue, and umbilical cord were pre-conditioned with PPARβ/δ agonist as previously described. RNA was isolated and quantified as described. OD 260/230 ratios were around 1.8 and 2.0.

### Preparation of the sequencing library

The 3′seq-RNA profiling (3′ SRP) was performed as described by McClure et al.^26^ Samples were randomly plated and diluted to a concentration of 2.5 ng/μL to construct the library. The library was prepared from 10 ng of total RNA in 4 µL. The mRNA poly-A tails were tagged with universal adapters, well-specific barcodes, and unique molecular identifiers (UMIs) during template-switching reverse transcriptase (Thermo Scientific^™^, EP0751, Maxima H Minus Reverse Transcriptase). Barcoded cDNAs from 96 samples were then pooled and purified using a Zymo kit. cDNAs were treated with exonuclease I (NEB, M0293S) before being amplified (12 cycles) and fragmented using a transposon-fragmentation approach that enriched for 3′ ends of cDNA. The fragments were size controlled on a 2200 TapeStation system (Agilent Technologies) to form a library of 350-800 bp in length. Finally, the library was validated with fragments of approximately 484 pb length and with 38.5 nM of properly tagged cDNA.

### Sequencing and primary analysis

Sequencing and primary analysis were performed according to Charpentier et al.^27^ Briefly, the library was sequenced on a NovaSeq 6000 using NovaSeq 6000 SP Reagent Kit 100 cycles (#20 027 464, Illumina) with 17*-8–105* cycle reads (* addition of one cycle according to Illumina’s recommendation). Bioinformatics steps were performed using a Snakemake pipeline. Sample sequences were demultiplexed using a python script, and raw paired-end fastq files were transformed into a single-end fastq file for each sample and aligned on the Ensembl transcriptome hg38 using bwa.

### Differential expression analysis

Aligned reads were parsed, and UMIs were counted for each gene in each sample to create an expression matrix containing the absolute abundance of mRNAs across all samples. Reads aligned on multiple genes or containing more than 3 mismatches with the reference were excluded. Prior to differential expression analysis, genes with low counts (total UMIs per gene ≤ 10) were filtered out to ensure robust results. Differential expressed analysis was then conducted using the DeSeq2 package in RStudio, following the standard workflow.^28^ The expression matrix was normalized, and differentially expressed genes (DEGs) were identified using adjusted *P*-value < 0.1 and log2(fold-change) > 0.5. Finally, volcano plots were created with EnhancedVolcano package.^29^

### Statistical analysis

GraphPad Prism v10.1.1 software (GraphPad, USA) was used for the presentation of the results and statistical analysis. To determine statistical differences, a paired *t*-test was used to compare 2 conditions, while a two-way analysis of variance was conducted for the *in vitro* experiments comparing multiple conditions. For *in vivo* studies, an unpaired non-parametric Mann–Whitney test was selected; *P ≤ .05, **P ≤ .01, ***P ≤ .001, and ****P ≤ .0001; ns: not significant.

## Results

### Enhanced capacity of PPARβ/δ primed MSCs vs naïve MSC to protect cartilage from degradation and to limit OA progression in a collagenase-induced OA murine model

**Figure 1.**
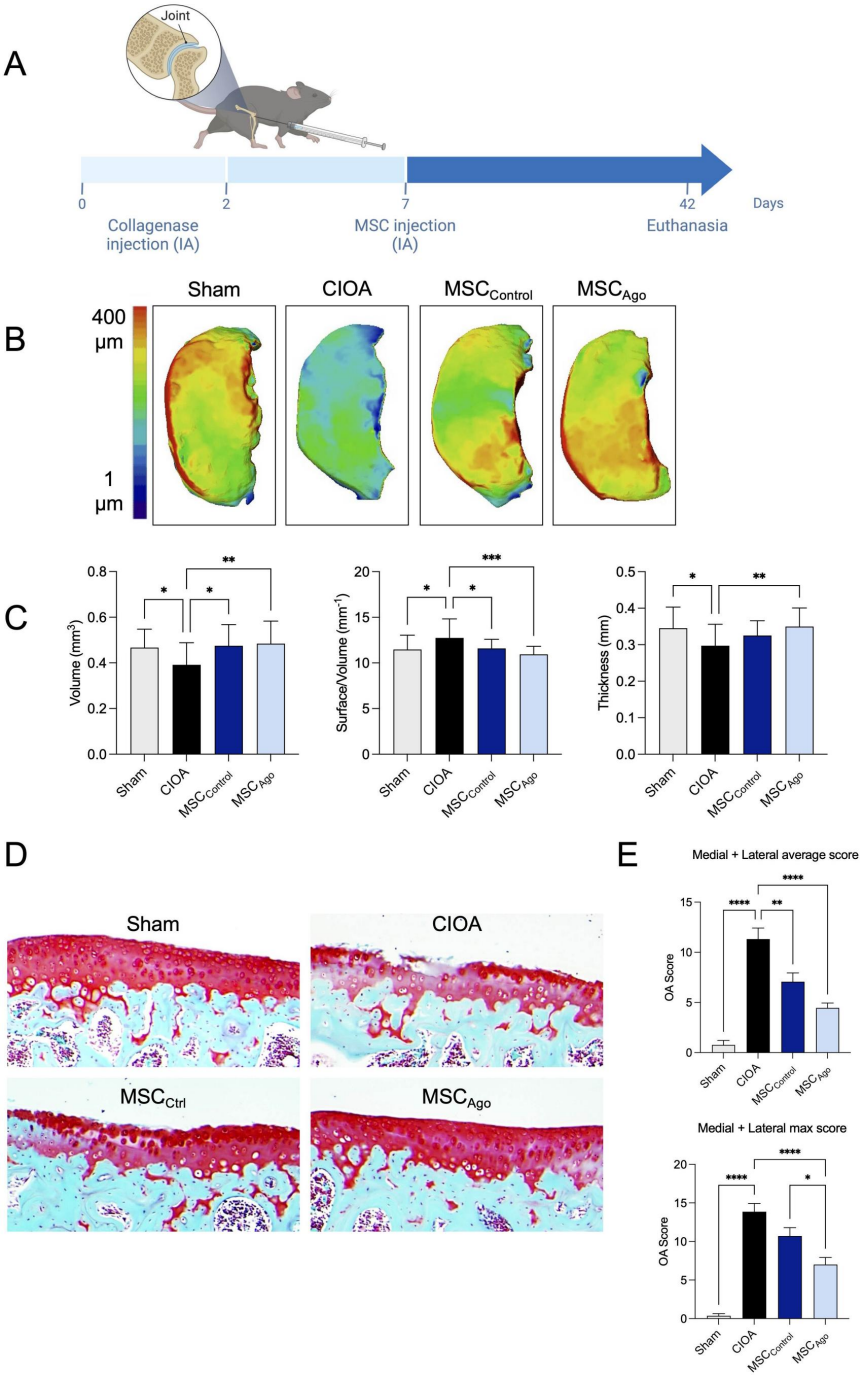
Activation of PPARβ/δ enhances therapeutic efficacy of murine mesenchymal stromal cell (MSC) in a collagenase-induced osteoarthritis (CIOA) murine model *in vivo*. (A) On Day 0 and 2, mice received intra-articular injection (IA) of 1 unit of type VII collagenase. On Day 7, 2.5 × 10^5^ MSC control (MSC_Control)_ or pretreated with 1 µm PPAR agonist (MSC_Ago_) were IA injected. After 42 days, mice were euthanized and knee joints were harvested and fixed for histological analysis. (B) Representative 3D reconstructed images of medial plateau of articular cartilage post confocal laser scanning microscopy analysis. (C) Quantitative histomorphometric evaluation of 3D reconstructed images displaying volume, surface degradation and thickness of articular cartilage. (D) Representative histological sections of tibial articular cartilage stained with Safranin-O-Fast green. (E) Osteoarthritis (OA) average and maximal score measured in both medial and lateral tibia plateaus. Data are presented as mean ± SD; *n* = 10-13 mice per group; *P < .05, **P < .01, ***P < .001, ****P < .0001. Created in BioRender. Djouad, F. (2026) https://BioRender.com/b2xcplu.

We assessed the therapeutic effects of naïve (MSC_control_) versus PPARβ/δ agonist-primed MSC (MSC_Ago_) in an OA model previously established to demonstrate MSC efficacy.^24^ MSCs (2.5 × 10^5^) were injected on Day 7 post-OA induction, with cartilage integrity evaluated at Day 42 using confocal laser scanning microscopy to analyze the lateral and medial tibial plateau (Figure 1A). MSC_Ago_ significantly improved key cartilage parameters including the volume, the degradation (surface/volume ratio), and the thickness, MSC_control_ showed improvement in volume and degradation metrics only (Figure 1B and C). Histological assessment and OA scoring confirmed that both MSC types reduced cartilage degradation, though PPARβ/δ-primed MSCs provided superior protection (Figure 1D and E). These findings collectively indicate that MSCs primed with PPARβ/δ agonist exhibit superior therapeutic efficacy in experimental OA models compared to control MSCs.

### Immunoregulatory properties of PPARβ/δ-primed MSCs are comparable to those of naïve MSCs

**Figure 2.**
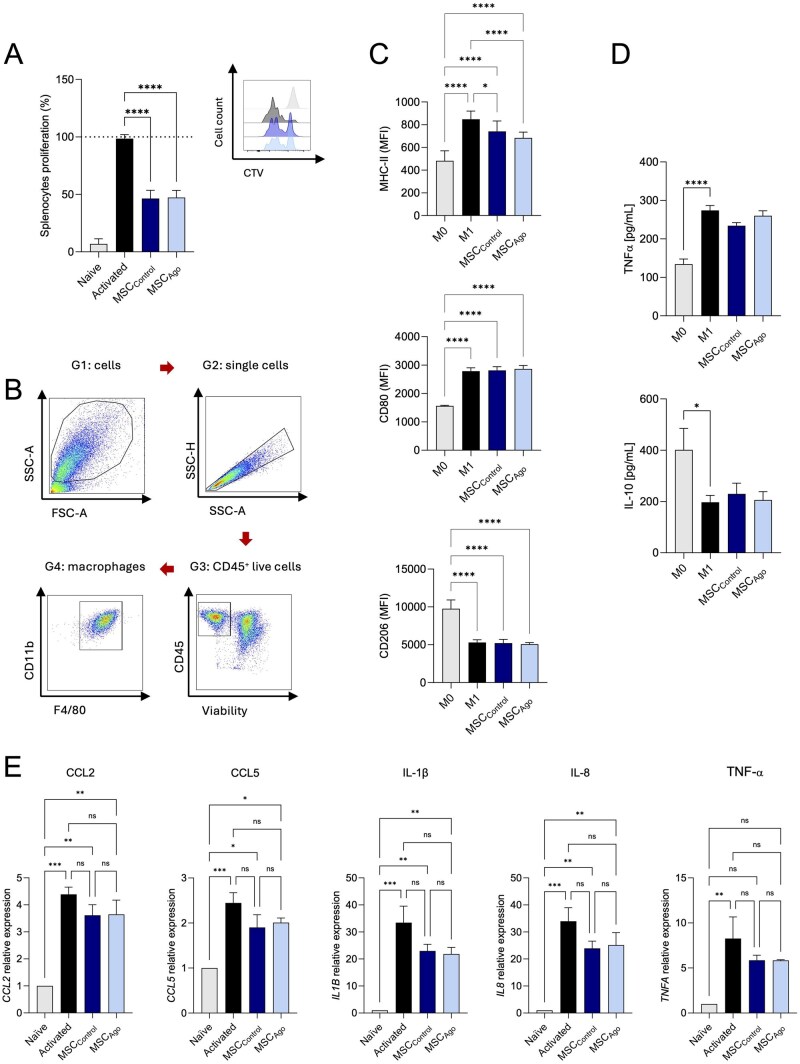
Treatment with peroxisome proliferator-activated receptor (PPAR) agonist does not affect the immunomodulatory capacity of mesenchymal stromal cells (MSCs). (A) Concanavalin A-activated splenocytes proliferation and representative histogram. (B) Gating strategy for macrophages analysis. Cells were first selected based on their size and granularity, then double events were excluded, as well as dead cells, finally cells were gated on CD45^+^CD11b^+^F4/80^+^ population, considered as differentiated macrophages. (C) The median fluorescence intensity (MFI) of pro-inflammatory-associated markers (CD80 and MHC-II) and anti-inflammatory-associated marker (CD206) was analyzed. Data are presented as the mean fluorescence intensity ± SEM; *n* = 3. (D) Supernatants from macrophages cultures were analyzed by ELISA to determine the presence of prof-inflammatory (tumor necrosis factor alpha [TNFα]) and anti-inflammatory (interleukin-10 [IL-10]) mediators. (E) Relative mRNA expression of inflammatory/chemotactic genes (*CCL2*, *CCL5*, *IL1B*, *IL8*, and *TNFA*) on SW982 synovial cells was quantified by RT-qPCR and normalized to *ACTB*. Data are shown as fold change relative to the Naive condition and represent mean ± SEM from *n* = 5 independent experiments. *P < .05, *P < .01, ***P < .001, ****P < .0001, ns: not significant.

MSCs represent promising therapeutic candidates for OA treatment due to their diverse properties, particularly their immunomodulatory effects. Indeed, MSCs can alter the inflammatory cytokine environment and influence immune cell responses in the joint, primarily through the release of paracrine factors and EVs.^30–33^ Thus, we investigated whether priming MSCs with PPARβ/δ agonist could modify their immunosuppressive properties. To address this issue, MSC_control_ and MSC_Ago_ were co-cultured with concanavalin A-activated splenocytes at a 1:10 ratio for 48 hours (Figure S1A, see online supplementary material for a color version of this figure). First, we confirmed the immunosuppressive capabilities of naïve MSCs on cell trace violet (CTV)-labeled splenocytes activated with concanavalin A. While MSC_Ago_ significantly inhibited the proliferation of activated splenocytes, we did not observe any difference in the immunosuppressive effects between MSC_control_ and MSC_Ago_ (Figure 2A).

Given that several studies have shown a substantial increase in macrophage populations in OA synovial membranes and their associated pro-inflammatory characteristics and recognizing the robust immunoregulatory effects of MSCs on macrophages, we explored the impact of PPARβ/δ priming on this later MSC function. To that end, murine primary macrophages were differentiated and activated to induce a pro-inflammatory M1-like phenotype. Then, MSC_control_ or MSC_Ago_ were co-cultured with activated macrophages at 1:10 ratio for 24 hours, and macrophage phenotype was assessed by flow cytometry (FACS) and ELISA (Figure S1B, see online supplementary material for a color version of this figure). For FACS analysis, cells were gated on CD45^+^CD11b^+^F4/80^+^ populations (Figure 2B). We found that both MSC_control_ or MSC_Ago_ significantly reduced MHC-II expression in macrophages as compared to pro-inflammatory M1-like macrophages cultured alone. However, both types of MSCs had no effect on CD80 and CD206 expression levels in macrophages (Figure 2C). Additionally, when we examined the capacity of macrophages to produce TNF and IL-10, we noticed that both MSC_control_ and MSC_Ago_ did not alter their secretory activities (Figure 2D). Collectively, these results indicate that PPARβ/δ priming of MSCs did not alter their immunosuppressive effects on both activated splenocytes and pro-inflammatory macrophages, maintaining immunomodulatory properties comparable to MSC_Control_.

To assess whether PPARβ/δ priming enhances MSC-mediated regulation of synovial inflammation, SW982 synoviocytes were activated with IL-1β (5 ng/mL) for 6 hours and then co-cultured in transwell with either MSC_Ctrl_ or MSC_Ago_ at a 1:5 MSC: SW982 ratio (Figure S1C, see online supplementary material for a color version of this figure). IL-1β stimulation robustly increased the expression of inflammatory/chemotactic genes (*CCL2, CCL5, IL1B, IL8,* and *TNFA*) compared with non-activated cells. Co-culture with MSCs modestly attenuated this IL-1β–induced transcriptional response for *CCL2, CCL5, IL1B*, and *IL8* but not for *TNFA*. However, PPARβ/δ priming did not significantly enhance these anti-inflammatory properties, as no differences were observed between control and primed MSCs (Figure 2E).

### Enhanced resistance of PPARβ/δ-primed MSCs to oxidative stress

**Figure 3.**
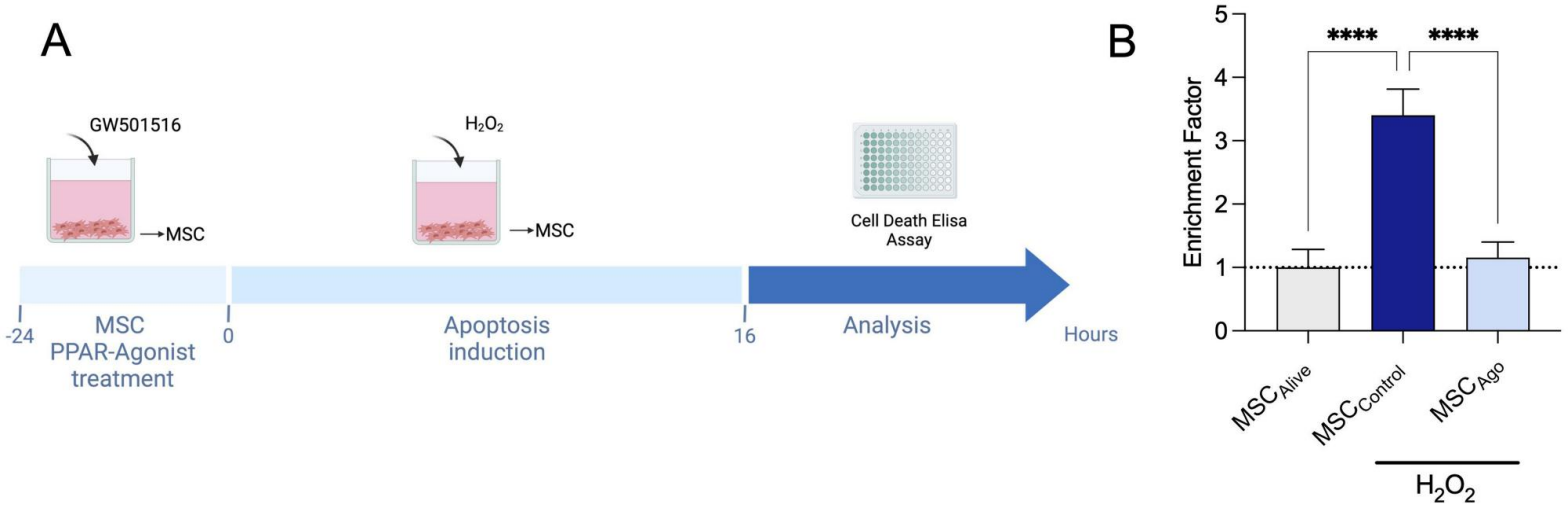
PPARβ/δ priming protects mesenchymal stromal cells from H_2_O_2_-induced apoptosis. (A) MSC_Control_ or MSC_Ago_ were exposed to H_2_O_2_ for 16 hours, then apoptosis was measured by DNA fragmentation assay. (B) Bar plots representing specific DNA fragmentation after H_2_O_2_ exposure results are expressed as fold change (enrichment factor) relative to untreated cells (MSC_alive_), indicated by the dotted line. Data are presented as the mean ± SEM; *n* = 5. ****P < .0001. Created in BioRender. Djouad, F. (2026) https://BioRender.com/b2xcplu.

We recently demonstrated that MSC pre-treated by the PPARβ/δ agonist GW0742 are protected from apoptosis induced by H_2_O_2_ stress.^22^ Since elevated oxidative stress contributes significantly to chronic inflammation in OA,^34–36^ we wanted to test whether PPARβ/δ priming with GW501516 could protect MSC stressed by H_2_O_2_. Therefore, to study the role of PPARβ/δ priming on MSC challenged by OA stress environmental conditions, cultured MSC_control_ and MSC_Ago_ were submitted to an OA-simulated stress *in vitro* using H_2_O_2_ (100 µM) for 16 hours (Figure 3A). Apoptosis quantification was assessed by the mean of specific DNA fragmentation measurement for the different groups of treatment. To determine the role of PPARβ/δ on the resistance of MSC to H_2_O_2_, we pre-treated them with PPARβ/δ agonist (1 µM) for 24 hours before the H_2_O_2_-stress induction. While MSC_control_ exposed to H_2_O_2_ stress exhibited a 3.47-fold increase in apoptotic response, the apoptotic response in MSC_Ago_ remained comparable to that of non-stressed MSCs (MSC_Alive_) (Figure 3B). Altogether, these results revealed that MSC activated for PPARβ/δ were protected from apoptosis induced by H_2_O_2_ stress.

### Increased anti-apoptotic effects of PPARβ/δ-primed MSC on chondrocytes exposed to an oxidative stress

**Figure 4.**
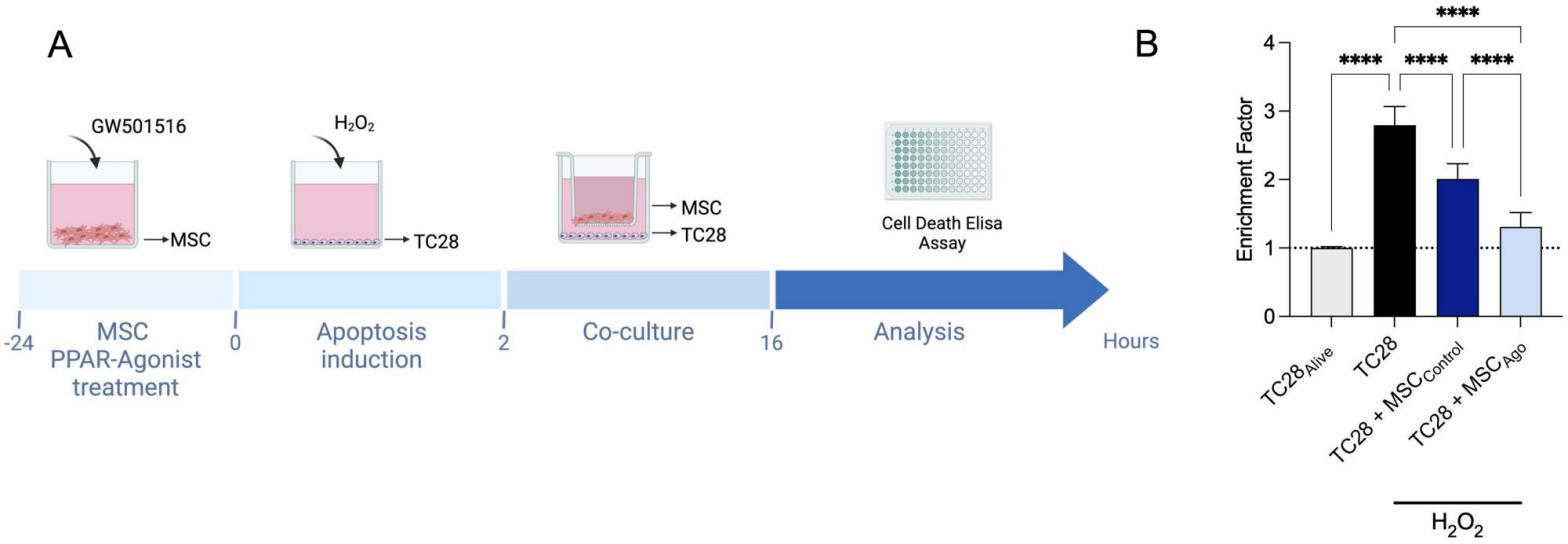
MSC PPAR_Ago_ exhibit an enhanced anti-apoptotic effect on chondrocytes. (A) TC28a2 human chondrocytes were exposed to 100 µm H_2_O_2_ for 2 hours to induce apoptosis, then MSC_Control_ or MSC_Ago_ were co-cultured in transwell inserts at 1:2 ratio for an additional 14 hours (16 hours in total). Apoptosis in chondrocytes was quantified using a cell death detection ELISA. (B) Bar plots show specific DNA fragmentation after H_2_O_2_ exposure. Results are expressed as fold change (enrichment factor) relative to untreated cells (TC28_Alive_), indicated by the dotted line. Data are presented as mean ± SEM (*n* = 5). ****P < .0001. Created in BioRender. Djouad, F. (2026) https://BioRender.com/b2xcplu.

To investigate the role of PPARβ/δ priming on the anti-apoptotic properties of MSCs on stressed chondrocytes, we used transwell systems to co-culture MSC_Control_ and MSC_Ago_ with cells of the TC28a2 human chondrocyte cell line. The experimental protocol consisted of exposing TC28a2 chondrocytes to 100 µM of H_2_O_2_ for 2 hours before co-culturing them for additional 14 hours with either MSC_Control_ or MSC_Ago_ (Figure 4A). Our initial findings indicated that treatment with 100 µM of H_2_O_2_ for 16 hours significantly increased apoptosis in TC28a2 chondrocytes by 2.79-fold compared with unchallenged cells (TC28_Alive_) (Figure 4B). However, co-culture with either naive or PPARβ/δ-primed MSCs significantly protected the chondrocytes from H_2_O_2_-induced stress. Notably, the anti-apoptotic effect was significantly enhanced in chondrocytes co-cultured with PPARβ/δ-primed MSCs compared to those co-cultured with MSC_Control_, showing a decrease in the enrichment factor from 2.0- to 1.3-fold increase, respectively. The results demonstrate that PPAR activation enhances the protective effects of MSC on oxidative stress-induced apoptosis in chondrocytes.

### Highest responsiveness of *Angptl4* gene upon PPARβ/δ priming, regardless of the species and the sources of the MSCs

**Figure 5.**
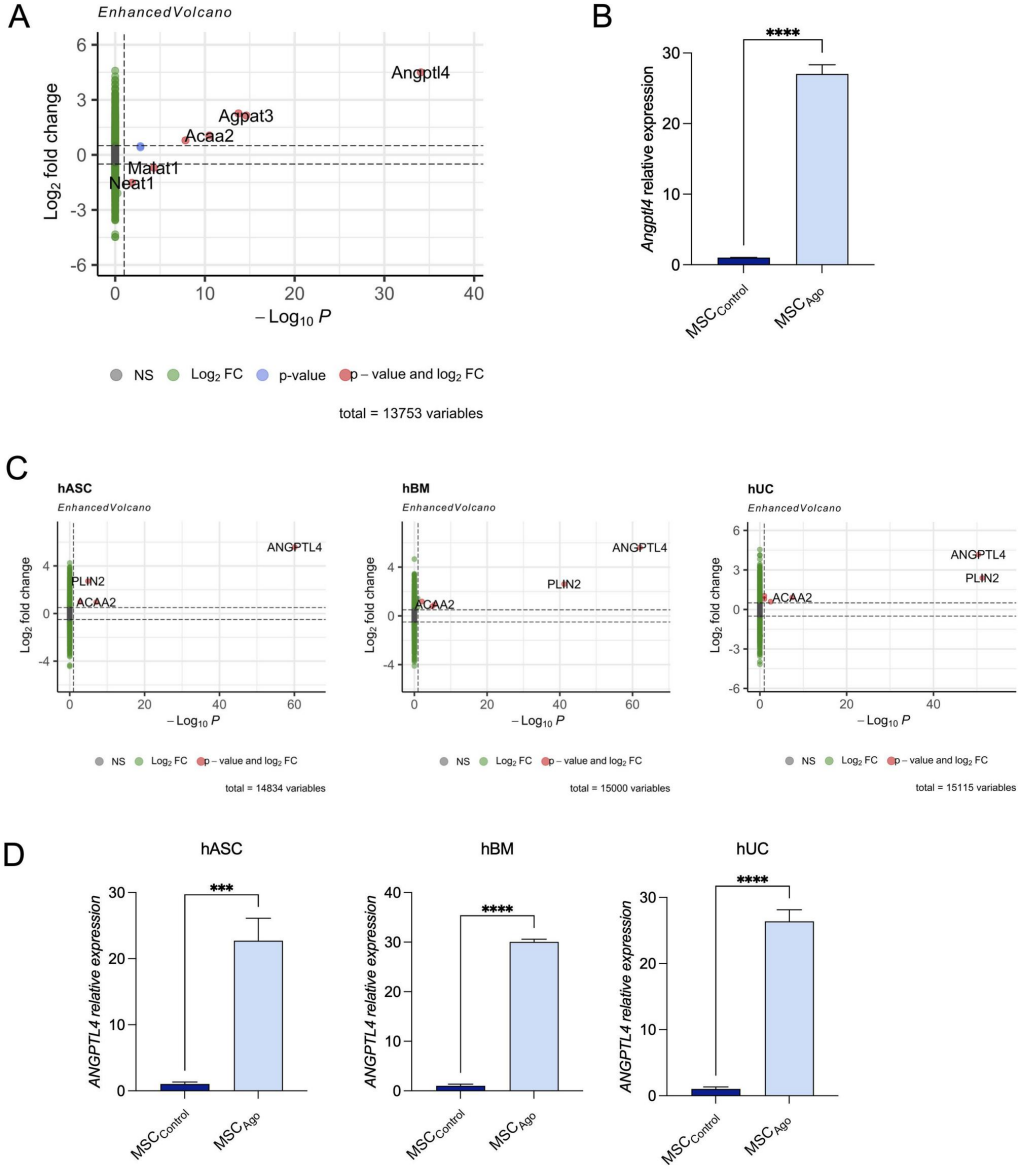
Mechanisms involved in the enhanced properties of PPARβ/δ-primed MSCs. (A) Volcano plot of the 13 753 variables identified by RNA seq analysis on murine MSC. Volcano plot shows the significance (non-adjusted *P*-value) converted to –Log10(*P*-value) versus the fold change converted to Log2(fold change). For the analysis, a gene was considered significantly differentially expressed when *P*-value ≤ 0.05 and absolute fold change  ≥ 1.5. (B) The expression of *Angptl4* in murine MSC was validated by qRT-PCR. (C) Volcano plots of human MSC derived from bone marrow (hBM), adipose tissue (hASC), and umbilical cord (hUC). (D) The expression of *ANGPTL4* was validated by qRT-PCR in human MSC derived from hBM, hASC, and hUC. Data are presented as the mean ± SD; *n* = 4. ***P < .001; ****P < .0001.

We then explored the mechanisms underlying the enhanced chondroprotective and therapeutic properties of PPARβ/δ-primed MSCs in OA. To do this, we used 3′ SRP, which uses sample multiplexing and mRNA molecular indexing to facilitate genome-wide transcriptional profiling of naive and PPARβ/δ-primed MSCs. Visualization of the RNA-seq data using a volcano plot revealed that the most significantly overexpressed gene in response to PPARβ/δ priming was *Angptl4* in murine PPARβ/δ-primed MSCs compared with naive MSCs (Figure 5A). This substantial increase in *Angptl4* expression was validated by qRT-PCR (Figure 5B). *Angptl4* is well-stablished downstream target gene of the PPAR pathway.^37^ To determine whether the PPARβ/δ agonist-induced increase in *Angptl4* observed in bone marrow-derived murine MSCs also occurs in clinically relevant MSC sources, we examined the effects of PPARβ/δ priming in human MSCs derived from adipose tissue (hASC), bone marrow (hBM), and umbilical cord (hUC). Our results indicate that PPARβ/δ priming also significantly increased *ANGPTL4* expression levels in all 3 human MSC sources (Figure 5C and D). These findings suggest that PPARβ/δ agonist increases *ANGPTL4* transcript expression in MSC independently of species and cell sources.

### PPARβ/δ enhances MSC resistance to oxidative stress and their anti-apoptotic effect on chondrocytes under H_2_O_2_ stress in an *Angptl4*dependent manner

**Figure 6.**
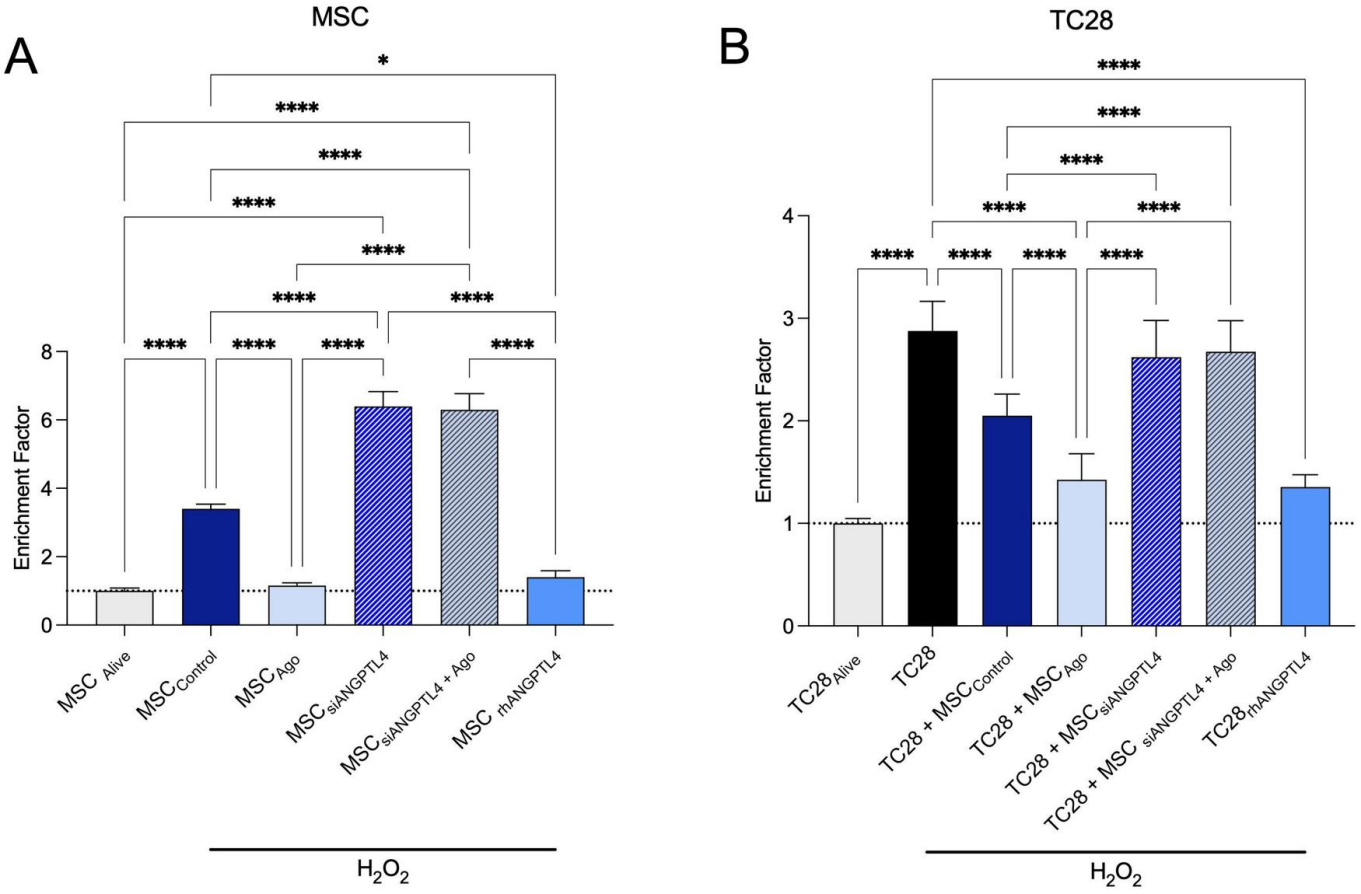
ANGPTL4 mediates the antiapoptotic effect induced by PPARβ/δ agonist priming. Mesenchymal stromal cell (MSC) were silenced with specific siRNA against *Angptl4* and primed or not with peroxisome proliferator-activated receptor (PPAR) agonist. (A) MSCs pre-treated or not were exposed to H_2_O_2_ for 16 hours, and then apoptosis was quantified using a Cell Death ELISA kit. (B) Chondrocytes were exposed to H_2_O_2_ for 2 hours to induce apoptosis, then MSC were co-cultured in transwell inserts at 1:2 ratio for an additional 14 hours (16 hours in total). Bar plots show specific DNA fragmentation after H_2_O_2_ exposure. Results are expressed as fold change (enrichment factor) relative to untreated cells (dotted line). Data are presented as the mean ± SEM; *n* = 5. *P < .05; ****P < .0001.

To investigate whether *Angptl4* acts as a critical signaling molecule in enhancing the beneficial effects of MSCs, we used small interfering RNA (siRNA) to silence *Angptl4* in murine MSCs. After confirming the successful knockdown of *Angptl4* in both MSC_control_ and MSC_Ago_ (Figure S2, see online supplementary material for a color version of this figure), we exposed these cells to oxidative stress using H_2_O_2_ at a concentration of 100 µM for 16 hours. We then measured apoptosis by quantifying specific DNA fragmentation across different treatment groups. Our findings showed that H_2_O_2_ exposure at 100 µM significantly induced apoptosis in MSCs compared to unchallenged controls (MSC_Alive_), resulting in a 3.4-fold increase in apoptosis. In contrast, MSC_Ago_ were fully protected from H_2_O_2_-induced apoptosis with levels comparable to those of MSC_Alive_ (Figure 6A). However, this protective effect was completely abolished in all *Angptl4*-silenced MSCs in the absence or in the presence of PPARβ/δ priming, as evidenced by apoptosis rates rising to a 6.39- and 6.29-fold increase, respectively. To confirm the role of ANGPTL4 in mediating the effects of PPARβ/δ agonist priming, H_2_O_2_-stressed MSC were cultured in the presence of recombinant ANGPTL4. Notably, treatment with ANGPTL4 provided a level of protection against H_2_O_2_ comparable to that observed with PPARβ/δ priming (1.4-fold increase) (Figure 6A).

To further examine the role of *Angptl4* in the anti-apoptotic effects of MSC_control_ or MSC_Ago_ on H_2_O_2_-stressed chondrocytes, we silenced *Angptl4* in both types of MSC before co-culturing them with H_2_O_2_-stressed TC28a2 human chondrocytes in transwell systems. Our data showed that *Angptl4*-silenced MSC, whether naive or PPARβ/δ-primed, lost their protective effect on H_2_O_2_-stressed TC28a2 chondrocytes, resulting in apoptosis rates rising 2.6-fold and 2.67-fold increases, respectively, displaying apoptosis rates similar to those of TC28a2 challenged cells (2.87-fold increase). In contrast, MSC_control_ or MSC_Ago_ expressing *Angptl4* significantly reduced chondrocyte apoptosis, a protective effect further enhanced in PPARβ/δ-primed MSCs (2.05-fold increase and 1.42-fold increases, respectively) (Figure 6B). Of note, recombinant ANGPTL4 also effectively protected TC28a2 chondrocytes under oxidative stress, achieving protection comparable to that of PPARβ/δ-primed MSCs (1.35-fold increase and 1.42-fold increase, respectively) (Figure 6B). These results indicate that PPARβ/δ activation enhanced the anti-apoptotic potential of MSCs in protecting chondrocytes exposed to oxidative stress *in vitro*, and this effect was dependent on *Angptl4* expression.

## Discussion

Our results demonstrated that both control MSCs or pre-conditioned with specific PPARβ/δ agonist were able to treat the effects of OA in an *in vivo* collagenase-induced OA model. This beneficial effect was reflected into enhanced cartilage preservation, as evidenced by CL-SM analysis and histological analysis, with reduced loss of cartilage volume and thickness, and lower OARSI scores, reaching levels comparable to those observed in sham controls, for both control and preconditioned MSCs. Interestingly, MSC primed with PPARβ/δ agonist displayed an enhanced protective effect on cartilage compared to untreated MSC as evidenced by safranine-O-fast green staining. These data indicate that PPARβ/δ priming enhances the chondroprotective potential of MSCs *in vivo*, beyond benefit provided by naïve MSCs.

Given that OA is a disease characterized by cartilage degradation and chondrocyte apoptosis, along with an inflammatory component derived from synovial cells and infiltrating macrophages,^1^^,^^38^^,^^39^ we first evaluated whether treatment of MSCs with PPARβ/δ agonist could improve MSC-based therapy by modulating classical immune regulatory mechanisms. In line with their well-established immunoregulatory properties, naïve MSCs reduced splenocyte proliferation and slightly modulated macrophage activation, as well as partially attenuated IL-1β-induced inflammatory/chemotactic gene expression in SW982 synoviocytes. However, PPARβ/δ priming did not significantly enhance these effects and MSC_Ago_ and MSC_control_ exhibited comparable immunomodulatory capacities. Together, these findings indicate that, under our experimental conditions, PPARβ/δ activation does not confer a generalized gain-of-function in terms of MSC immunosuppression.

Instead, our data highlight a prominent role of PPARβ/δ priming in reinforcing cyto-protective and anti-apoptotic functions of MSCs. Oxidative stress is a major contributor to cartilage degeneration and cell death in OA, contributing to disease progression.^40^ In our *in vitro* assays, PPARβ/δ-primed MSCs were more resistant to H_2_O_2_-induced cell death and more effectively preserved the viability of oxidatively stressed chondrocytes than naïve MSCs. This dual effect is particularly relevant in the hostile osteoarthritic joint microenvironment and may increase the time window during which MSCs can exert cytoprotective and pro-regenerative properties.

Transcriptomic analysis allowed us to identify angiopoietin-like 4 (*Angptl4*) as one of the most significantly upregulated genes in PPARβ/δ-primed MSCs compared to untreated cells. Importantly, ANGPTL4 upregulation was consistently observed across murine MSCs and human MSC derived from different tissues, including adipose tissue, bone marrow, and umbilical cord, supporting that ANGPTL4 induction is a robust response to PPARβ/δ activation conserved across species and MSC sources. ANGPTL4 has been shown to confer a protective effect against apoptosis in several cell types. In MSCs, exogenous ANGPTL4 prevents apoptosis induced by hypoxia and serum deprivation, and enhances paracrine functions by upregulating the secretion of vascular endothelial growth factor (VEGF), basic fibroblast growth factor (bFGF), hepatocyte growth factor (HGF), and insulin-like growth factor (IGF).^41^ Notably, all these factors have been also described to exert anti-apoptotic effects in different cell types, such as endothelial cells, cancer cells; osteoblasts, hepatocytes, and cardiomyocytes.^42–49^ In line with these reports, other studies in tumor cells indicate that ANGPTL4 mediates antiapoptotic effects via activation of PI3K/AKT signaling pathway in an autocrine manner.^50^^,^^51^ In our study, functional experiments demonstrated that ANGPTL4 is required for the anti-apoptotic effect of PPARβ/δ priming: *Angptl4* silencing abrogated the resistance of MSC_Ago_ to oxidative stress and diminished their ability to protect chondrocytes, whereas recombinant ANGPTL4 partially mimicked the protective effect of primed MSCs.

Several strategies have been proposed to improve MSC efficacy in OA, including hypoxic preconditioning, inflammatory licensing, pharmacological modulation of metabolism, and genetic engineering.^52^ Although these approaches have yielded promising results, their biological outputs may vary substantially depending on donor characteristics and manufacturing conditions, which can complicate standardization and inter-study reproducibility. In contrast, PPARβ/δ-driven molecular priming relies on a defined nuclear receptor that can be selectively targeted pharmacologically, together with a well-characterized agonist, to induce a reproducible pro-survival program driven by ANGPTL4. By focusing on cell-intrinsic resistance to oxidative stress and direct chondroprotection, rather than aiming to amplify immunosuppression, this strategy addresses a major limitation in MSC therapy for OA: the vulnerability of both MSCs and chondrocytes to oxidative damage within the diseased joint.

Our study has several limitations that should be considered when extrapolating these findings to human disease. First, the CIOA model, while widely used, does not fully reproduce the complexity and heterogeneity of human OA. Recent detailed characterization of CIOA has shown dose-dependent progression of cartilage degeneration and bone remodeling, with synovial changes emerging at later stages, and mechanistic links to collagenase-driven joint instability that compress the disease timeline compared with human OA.^53^ Accordingly, validation in complementary OA models (including surgically induced and metabolic OA) will be important prior to broader translation. Second, MSC heterogeneity remains a key translational issue; while most experiments used a single murine MSC line, the conserved induction of ANGPTL4 across multiple human MSC sources and donors supports the robustness of the priming response, yet broader testing across clinical-grade lots will be required. Third, outcomes were assessed at day 42, which does not address long-term durability or safety, warranting extended follow up with dedicated safety endpoints. Finally, we did not assess pain-related behaviors; given the recognized contribution of neuroimmune interactions to OA pain,^54^^,^^55^ future studies should integrate standardized behavioral readouts to determine whether the structural and cytoprotective benefits of MSC_Ago_ translate into functional improvement.

Together, our results suggest that the increased *Angptl4* expression in PPARβ/δ-primed MSCs may act in an autocrine fashion to protect MSCs from H_2_O_2_-induced oxidative stress and exert a paracrine effect on target chondrocytes. Furthermore, this increased expression of *Angptl4* induced by PPARβ/δ priming may improve MSC survival and function indirectly by promoting the release of other anti-apoptotic factors, which could benefit MSCs and other cell types.

## Conclusions

This study shows that PPARβ/δ priming enhances the therapeutic efficacy of MSCs in OA by significantly enhancing their resistance to oxidative stress and improving their anti-apoptotic effects on chondrocytes. Mechanistically, we found that PPARβ/δ priming upregulates the expression of *Angptl4* which is involved in the enhanced protective benefits of MSCs associated with PPARβ/δ priming. *ANGPTL4* expression was significantly upregulated in PPARβ/δ-primed MSCs across species and MSC sources, suggesting a conserved response to PPARβ/δ activation. ANGPTL4 likely acts in an autocrine manner to protect MSCs from oxidative stress and in a paracrine fashion to shield chondrocytes from apoptosis. These findings suggest that targeting PPARβ/δ could be a promising strategy to enhance MSC-based therapies for OA by improving cell survival, chondroprotection, and overall efficacy in cartilage preservation. Future research should explore the clinical translation of PPARβ/δ-primed MSCs to evaluate their potential benefits in OA patients.

## Supplementary Material

szag053_Supplementary_Data

## Data Availability

High-throughput sequencing data have been submitted in the Gene Expression Omnibus (GEO) database under the GEO accession number GSE283154. Additional data supporting the findings of this study are available from the corresponding author upon request.
