# Supplementary material for: PPARβ/δ priming enhances mesenchymal stromal cell anti-apoptotic properties on chondrocytes via ANGPTL4 and improves their therapeutic effects in osteoarthritis
Source: Stem Cells Transl Med. 2026 Aug 5;15(8):szag053. doi: 10.1093/stcltm/szag053 (PMC13441182; doi:10.1093/stcltm/szag053)
Supplement: Supplementary file 1 [file szag053_supplementary_data.docx]

**PPARβ/δ priming enhances MSC anti-apoptotic properties on chondrocytes via ANGPTL4 and improves their therapeutic effects in Osteoarthritis**

**Supplementary information**

**Running head:** PPARβ/δ priming boosts MSC chondroprotection

***Authors:***

Claudia Terraza-Aguirre ^1,4^, Rafael Contreras-López ^1^, Clara Bernard ^1,4^, Carolina Pradenas ^1^, Jholy De la Cruz ^1^, Audrey Barthelaix^1^, Catalina Andrea Asencio Barria ^1^ Gautier Tejedor ^1^, Cécile Boyer ^5^, Stéphanie Barrère-Lemaire ^2^, Christian Jorgensen ^1,3^, Mingxing Wei ^4^, Jerome Guicheux ^5^, Claire Vinatier ^5^, Farida Djouad ^1,3^

***Affiliations:***

^1^IRMB, Université de Montpellier, INSERM, Montpellier, France; ^2^IGF, Université de Montpellier, CNRS, INSERM, 141 rue de la Cardonille, 34094, Montpellier Cedex 5, France; ^3^Clinical Immunology and Osteoarticular Disease Therapeutic Unit, Department of Rheumatology, CHU Montpellier, 34095 Montpellier, France; ^4^ CellVax, Villejuif Bio Park, 1 mail du Professeur Georges Mathé - 94800 Villejuif, France; ^5^ Nantes Université, Oniris, CHU Nantes, INSERM, Regenerative Medicine and Skeleton, RMeS, UMR 1229, Nantes, France.

***Corresponding Author:***

**Farida DJOUAD**

INSERM, U 1183, IRMB, Hôpital Saint-Eloi, 80 avenue Augustin Fliche, 34295 Montpellier cedex 5, France - E-mail: farida.djouad@inserm.fr - Tel: +33 4 67 33 04 75

**Supplementary Figure 1. Representative workflows for immune and synovial cell assays. (A)** Immunosuppression assay. MSC_Control_ or MSC_Ago_ were cultured with total CTV-labeled splenocytes activated with concanavalin A for 48 hours. Then splenocytes proliferation was measured by FACS **(B)** Monocytes polarization assay. Bone marrow monocytes were isolated and differentiated for 4 days in the presence of M-CSF (20 ng/mL). Then, cells were activated with 250 ng/mL LPS and 20 ng/mL IFNγ for 24 hours to induce a pro-inflammatory M1-like phenotype. At day 5 control or pre-treated MSC were cultured for 24 hours with activated macrophages and phenotype was assessed by FACS. **(C)** Synoviocyte co-culture assay. Human SW982 synoviocytes were activated with 5 ng/mL IL-1β for 6 hours and subsequently co-cultured for 18 hours in transwell with either MSC_Ctrl_ or MSC_Ago_ at a 1:5 - MSC:SW982 ratio. Total RNA was then extracted from SW982 cells to quantify the expression of pro-inflammatory markers by qRT-PCR.

**Supplementary Figure 2. Validation of *Angptl4* silencing in murine MSC. (A)** *Angptl4* mRNA expression levels in murine MSCs assessed by qRT-PCR. A scrambled siRNA was used as a control. After silencing, cells were treated or not with PPAR agonist 1 µM for 24 hours. Data are presented as the mean ± SD; n=3. ****p<0.0001.
